# Characterization of terminated and withdrawn clinical trials for the treatment and prevention of oral mucositis

**DOI:** 10.1017/cts.2025.65

**Published:** 2025-04-10

**Authors:** Alex Reznik, Stephen Sonis, Alessandro Villa

**Affiliations:** 1 Medical Scientist Training Program, University of Miami Miller School of Medicine, Miami, FL, USA; 2 Department of Oral Medicine, Infection and Immunity, Harvard School of Dental Medicine, Boston, MA, USA; 3 Divisions of Oral Medicine and Dentistry, Brigham and Women’s Hospital and the Dana-Farber Cancer Institute, Boston, MA, USA; 4 Primary Endpoint Solutions, LLC, Waltham, MA, USA; 5 Oral Medicine, Oral Oncology, and Dentistry, Miami Cancer Institute, Baptist Health South Florida, Miami, FL, USA; 6 Department of Orofacial Sciences, University of California San Francisco, San Francisco, CA, USA

**Keywords:** Oral mucositis, radiation therapy, failed clinical trials, drug development, supportive care

## Abstract

**Purpose:**

Oral mucositis (OM) is a significant complication of cytotoxic cancer therapy and has no effective therapies. Unfortunately, the termination rate of clinical trials (CTs) testing potential OM interventions remains high. Here, we compared the characteristics of failed trials and matched completed trials to identify common features, which might inform better study design.

**Methods:**

CTs for the prevention/treatment of OM were identified using *ClinicalTrials.gov*. Failed (terminated or withdrawn) trials were evaluated for phase, type of cancer treatment (radiotherapy, chemotherapy, or chemo-radiotherapy), subject accrual, study type, number of clinical sites, intervention type, inclusion criteria, sponsor type, and reason(s) for failure. A secondary analysis of completed OM CTs that were individually matched to failed trials based on indication and phase or intervention type served as a control.

**Results:**

Failed OM CTs were more likely to have academic sponsorship (45.7% vs. 39.1%), nonrandomized design (19.6% vs. 4.3%), and lower mean subject accrual (27.8 subjects vs. 101.4 subjects) compared to completed trials. The leading reason for termination was recruitment/enrollment (37.9%). Recruitment/enrollment and safety/efficacy accounted for failure in 84.6% of phase II trials.

**Conclusion:**

Contrary to general CTs where safety/efficacy concerns predominate, our results suggest OM-related trial failures are associated with a broader list of challenges including recruitment/enrollment, funding/sponsorship, and investigator/site issues. OM CTs demand aggressive planning, funding, and careful selection of trial sites and sponsorship to assure timely subject recruitment and reduce the risk for early termination and withdrawal.

## Introduction

Oral mucositis (OM) is a common and devastating toxicity related to cancer therapy that is characterized by mucosal damage, which is typically associated with extensive and excessively painful ulcerations [1,2]. It is especially noteworthy among patients being treated with concomitant chemoradiation for head and neck cancers and those receiving myeloablative conditioning regimens in anticipation of hematopoietic stem cell transplants [3–5]. In addition to intense pain, severe oral mucositis (SOM; NCI-CTC or WHO grades of 3 or 4) is associated with nutritional deficiency, weight loss, cancer treatment dose de-escalation, treatment breaks, or early termination of treatment. The fiscal consequences of SOM are significant as patients often require supplemental nutrition via parenteral nutrition, increased unplanned office visits and emergency room use, and hospital admissions. Consequently, the incremental cost incurred by SOM is dramatic; it is noted to be in excess of $32,000 in the head and neck cancer (HNC) population and even more in hematopoietic stem cell transplantation (HSCT), where SOM extends hospital stays [6]. Despite its immense healthcare burden, effective treatment of severe OM remains elusive.

Neither the unmet clinical need nor the size of the potential global market (estimated to exceed $1 billion [US]) have gone unnoticed by independent investigators and the pharmaceutical industry. Interest in SOM as an indication has been solid and growing; however, SOM clinical trial (CT) success has been inconsistent, challenging the transition of innovative therapies to patients. This trend is not unique to SOM studies. Overall, the majority (>90%) of CTs fail [7]. Given this high rate, there have been many attempts to identify reasons for failure and have noted poor strategic planning [7], lack of efficacy (52%) and safety (24%) [8], and inadequate funding. Given the unique nature of a cancer supportive care indication and, particularly, SOM, we reasoned that a focused analysis of failed OM CTs might provide actionable insight for future studies. Our findings identify barriers to the development of OM therapies and can inform the design and funding of successful CTs.

## Materials and Methods

### Study Design

For the purposes of this study, we defined a failed CT as terminated or withdrawn prior to achieving the stated projected accrual. We identified failed CTs testing an intervention for OM secondary to radiotherapy (RT) and/or chemotherapy (CT) using *ClinicalTrials.gov* with the search term “oral mucositis” and study status (terminated, withdrawn) with start dates from 2000 to 2024. We restricted our search for OM secondary to cancer therapy; CTs indicated for the following conditions were excluded: inflammatory bowel diseases (Crohn’s disease, ulcerative colitis), toxic epidermal necrolysis, periodontitis, peri-implant mucositis, and stomatitis (recurrent aphthous, denture-related).

OM trials were assessed for phase, indication for cancer treatment (RT, CT, or chemo-radiotherapy [CRT]), subject accrual (actual, projected), number of clinical sites, intervention, inclusion criteria (HSCT and/or solid tumor), sponsor type (institution, government, academic, industry), and reason(s) for study termination or withdrawal. For trials with partial data omitted, a *PubMed* search was performed using the National Clinical Trial (NCT) number. For each failed (e.g. terminated or withdrawn) CT, we identified a completed OM CT on *ClinicalTrials.gov*.

As part of a secondary analysis, we identified “controls” (completed OM CTs) which were then individually matched to the failed CTs by indication for cancer treatment and phase of the trial, or indication for cancer treatment and intervention type. The characteristics of failed OM CTs and their matched controls are summarized in Table 1.

**Table 1.** Comparison of characteristics of failed and completed clinical trials

	Sponsor	Case	Control
*Phase I*	Industry	42.9% (3/7)	71.4% (5/7)
	Academic	28.6% (2/7)	28.6% (2/7)
	Governmental	14.3% (1/7)	–
	Private	14.3% (1/7)	–
	Collaborative group	–	–
	Single institution	85.7% (6/7)	85.7% (6/7)
	Multi-institution	14.3% (1/7)	14.3% (1/7)
	Inclusion criteria	Case	Control
	Solid tumor	71.4% (5/7)	71.4% (5/7)
	HSCT	14.3% (1/7)	14.3% (1/7)
	Both	14.3% (1/7)	14.3% (1/7)
	Indication	Case	Control
	RT + CT	57.1% (4/7)	71.4% (5/7)
	RT	14.3% (1/7)	14.3% (1/7)
	CT	–	14.3% (1/7)
	N/A	28.6% (2/7)	–
	Subject accrual	Case	Control
	Actual (% Accrual^a^)	1.8 (36.5%)	42.1 (99.1%)
	Projected	55.5	NR
*Phase II*	Sponsor	Case	Control
	Industry	31.6% (6/19)	57.9% (11/19)
	Academic	42.1% (8/19)	36.8% (7/19)
	Governmental	–	–
	Private	26.3% (5/19)	–
	Collaborative group	–	5.2% (1/19)
	Single-institution	89.5% (17/19)	68.4% (13/19)
	Multi-institution	10.5% (2/19)	31.6% (6/19)
	Inclusion criteria	Case	Control
	Solid tumor	78.9% (15/19)	73.7% (14/19)
	HSCT	10.5% (2/19)	10.5% (2/19)
	Both	10.5% (2/19)	15.8% (3/19)
	Indication	Case	Control
	RT + CT	73.7% (14/19)	57.9% (11/19)
	RT	10.5% (2/19)	36.8% (7/19)
	CT	15.8% (3/19)	5.2% (1/19)
	N/A	–	–
	Subject accrual	Case	Control
	Actual	24.2	91.7
	Projected	95.4	NR
*Phase III*	Sponsor	Case	Control
	Industry	20.0% (2/10)	50.0% (5/10)
	Academic	50.0% (5/10)	40.0% (4/10)
	Governmental	–	10.0% (1/10)
	Private	–	–
	Collaborative group	30.0% (3/10)	–
	Single-institution	70.0% (7/10)	80.0% (8/10)
	Multi-institution	30.0% (3/10)	20.0% (2/10)
	Inclusion criteria	Case	Control
	Solid Tumor	70.0% (7/10)	60.0% (6/10)
	HSCT	20.0% (2/10)	30.0% (3/10)
	Both	10.0% (1/10)	10.0% (1/10
	Indication	Case	Control
	RT + CT	40.0% (4/10)	30.0% (3/10)
	RT	20.0% (2/10)	40.0% (4/10)
	CT	30.0% (3/10)	30.0% (3/10)
	N/A	10.0% (1/10)	–
	Subject accrual	Case	Control
	Actual	60.3	206.3
	Projected	193.7	NR
**Total**	Sponsor	Case	Control
	Industry	26.1% (12/46)	54.3% (25/46)
	Academic	45.7% (21/46)	39.1% (18/46)
	Governmental	4.3% (2/46)	4.3% (2/46)
	Private	13.0% 6/46	–
	Collaborative group	10.9% (5/46)	2.2% (1/46)
	Single institution	82.6% (38/46)	73.9% (34/46)
	Multi-institution	17.4% (8/46)	26.1% (12/46)
	Inclusion criteria	Case	Control
	Solid tumor	69.6% (32/46)	67.4% (31/46)
	HSCT	19.6% (9/46)	19.6% (9/46)
	Both	10.9% (5/46)	13.0% (6/46)
	Indication	Case	Control
	RT + CT	54.3% (25/46)	54.3% (25/46)
	RT	15.2% (7/46)	28.3% (13/46)
	CT	23.9% (11/46)	17.4% (8/46)
	N/A	6.5% (3/46)	–
	Subject accrual	Case	Control
	Actual	27.8	101.4
	Projected	127.7	NR

Abbreviations: OM, oral mucositis; RT, radiotherapy; CT, chemotherapy; NR, not reported; N/A, not applicable.

a Percentage accrual (actual accrual/projected accrual) is listed in parenthesis.

This table includes sponsor, sponsor type, inclusion criteria, indication, mean, and projected subject accrual. The total includes trials with no phase (e.g. pilot and N/A). Table values have been calculated from trials with available data. Cases are defined as failed trials (either withdrawn or terminated), and secondary matching was performed to identify controls based on clinical trial phase and indication or intervention type.

### Data Analysis

Descriptive statistics were used to analyze reasons for failure, subject number and accrual, intervention, and sponsor types. We used a two-tailed Wilcoxon signed rank test to compare continuous variables (e.g. actual accrual, number of clinical sites) between cases (failed trials) and controls (completed trials). We used χ2 test to identify differences between cases and controls for the following: sponsor type. GraphPad Prism 10 and Microsoft Excel v16.86 were used for statistical analysis. Statistical significance was defined as *P* < 0.05.

### Data Availability

All data supporting results and analysis are publicly available on *ClinicalTrials.gov*.

### Ethics and Consent to Participate Statements

This work includes data retrieved from *ClinicalTrials.gov* which is publicly available. This work does not involve human subjects research.

## Results

### Study Characteristics and Reasons for Failure

Among the 621 OM-related CTs, we identified 46 failed trials (29 terminated, 17 withdrawn) for the treatment of RT and/or CT-induced OM. Of the failed trials, 73.9% (34/46) were randomized, 19.6% (9/46) were nonrandomized, and 6.5% (3/46) did not have multiple treatment arms. The majority of terminated trials (72.4%, 21/29) and withdrawn trials (76.5%, 13/17) were randomized, respectively. Of phase I trials, 75% (6/8) were nonrandomized and 87.5% (7/8) were open label. This includes all three terminated phase I trials, which were nonrandomized and open label. Of the failed trials, 45.7% (21/46) were open label: 44.8% (13/29) of terminated trials and 47.1% (8/17) of withdrawn trials. Table S1 outlines additional characteristics of failed OM trials, including phase, projected and actual patient accrual, percentage of projected accrual, number of clinical sites, and reasons for failure. For failed trials, the mean number of clinical study sites was 12.3 (range: 1–106) (Table S1). For completed trials, 80.4% (37/46) had a randomized study design, 4.3% were nonrandomized, and 15.2% did not have multiple treatment arms (N/A).

The most common reason for failure of terminated trials of all phases was recruitment/enrollment, with 37.9% (11/29) citing this reason. We found that 84.6% (11/13) of phase II trials were terminated due to either recruitment/enrollment or efficacy/safety (Table S1). The primary reasons for withdrawal were investigator/site issues (17.6%, 3/17), funding/sponsorship (17.6%, 3/17), and recruitment/enrollment (17.6%, 3/17).

### Subject Number and Accrual

Terminated trials enrolled 45.7% of subjects, and the mean actual accrual was 44.7 subjects (SD 62.5, 95% CI: 21.6-67.9) whereas mean projected accrual was 150.3 subjects (SD 103.1, 95% CI: 99.7-200.8) (Table S1). However, all withdrawn trials failed to enroll any subjects. Terminated phase III trials had the greatest actual accrual (mean 86.14 subjects, SD 85.6, 95% CI: 22.7–149.6) and projected accrual (Mean 222.7 subjects, SD 93.9, 95% CI: 147.6–297.8). The average number of failed trials per year from 2000 to 2024 was 1.84 (SD 1.4, 95% CI: 1.44–2.24). The number of failed trials did not increase during the COVID-19 pandemic from 2019 to 2023 (mean 1.80 trials per year, SD 1.2, 95% CI: 1.04–2.56). Failed trials had a lower mean actual accrual in comparison to completed trials (*P* < 0.05, 27.8 subjects vs. 101.4 subjects).

### Type of Intervention

Failed trials varied in the types of interventions for the treatment of OM. The most common interventions among terminated trials were dietary/nutritional (20.7%, 6/29) and small molecule (17.2%, 5/29). In contrast, of the withdrawn trials, 23.5% (4/17) were biologics, while dietary/nutritional, small molecule, and medical devices each accounted for 17.6% (3/17) of the trials.

### Sponsor Characteristics

**Figure 1.**
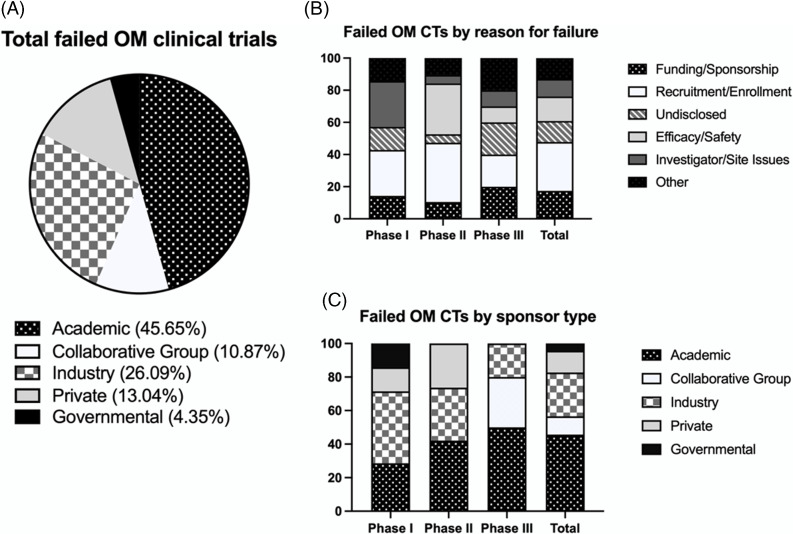
Summary of failed oral mucositis clinical trials by sponsorship and reason for failure. (A) Percentage of total failed oral mucositis (OM) clinical trials (CTs) by sponsor type. Failed trials are defined as terminated or withdrawn. (B) Percentage of failed OM CTs by reason for failure and CT phase. Total includes all 46 terminated or withdrawn CTs included in the study (phase I–IV and nonphase labeled studies). (C) Percentage of failed OM CTs by sponsor type and CT phase.

Failed trials varied by sponsor type; however, academic institutions accounted for 45.7% (21/46), followed by industry sponsors at 26.1% (12/46) (Figure 1, Table 1). Industry-sponsored trials were almost exclusively single-institution trials (91.7%, 11/12). Two-thirds (8/12) of industry-sponsored trials were supported by publicly traded companies, whereas the remaining one-third (4/12) were supported by private companies. In comparison to failed trials, a greater proportion of completed trials had industry sponsors across all phases (Table 1).

The sponsor type (single or multi-institution) was comparable between failed and completed trials (*P* = 0.312). Failed trials consisted of 82.6% (38/46) single-institution and 17.4% (8/46) multi-institution trials (Table 1). In contrast, completed trials consisted of 73.9% (34/46) single-institution and 26.1% (12/46) multi-institution trials. In comparison to failed trials, a greater number of matched completed trials were sponsored by the pharmaceutical industry: 54.3% (total: 25/46, public: 13/46, private: 12/46).

The inclusion criteria and indication for OM CTs trials were comparable between cases and controls (Table 1).

## Discussion

Management of OM remains challenging with only one FDA-approved agent, palifermin (Kepivance), for patients with hematological cancers undergoing high-dose conditioning regimens for HSCT [9], and no agents approved for RT-induced OM. A recent review compared the components of completed successful and failed Phase III trials intended to assess interventions for SOM in HNC patients being treated with concomitant chemoradiation [10]. In the current investigation, we purposefully included any phase study in which OM mitigation was the targeted efficacy endpoint.

Since 2000, 46 out of 621 OM-related CTs have been withdrawn or terminated. These studies have investigated a wide variety of therapies: biologics, small molecules, dietary/nutritional treatments, medical devices, analgesics/anesthetics, natural/herbal agents, oral care, and complementary/alternative therapy. We aimed to identify common features of failed CTs and contrast our findings with comparable features of completed studies with the goal of improving the number of successful trials and approved therapies for the treatment of OM.

Our findings indicate that a higher proportion of completed OM trials had industry sponsorship, randomized study design, and a higher mean actual accrual in comparison to matched failed trials. Our observations that a greater proportion of completed trials had industry sponsors were true across all phases. Furthermore, the overwhelming majority (91.7%) of industry-sponsored failed trials were single-institution trials. We further characterized sponsorship characteristics in failed OM trials and found a greater proportion were sponsored by academic institutions in comparison to matched controls. In support of our findings that failed OM trials had low subject accrual, the leading reason for failure among terminated trials was recruitment/enrollment (37.9%). For failed phase II OM trials, the vast majority (84.6%) cited recruitment/enrollment or safety/efficacy as reasons for failure.

Our results were not entirely consistent with a bulk analysis of CT data from 2010 to 2017 suggesting 40–50% of failures were attributed to lack of clinical efficacy, 30% to unmanageable toxicity, 10–15% to poor drug-like properties, and 10% due to poor strategic planning [7]. Importantly, such a comparison is not unexpected. Whereas we focused on terminated or withdrawn trials, more typical analysis of “failed” studies have assessed completed trials with unmet study endpoints. Thus, our findings are applicable to study design considerations aimed at assuring the completions of OM CTs, rather than risk mitigation around a specific agent (efficacy, safety). To that end, it seems clear that OM trials demand increased attention to particularities such as subject accrual, clinical site identification, and analysis of preclinical data.

Although the results of this study provide guidelines on the successful design of OM trials, we recognize several limitations. First, we were limited to publicly available information on *ClinicalTrials.gov*. Many studies did not provide all variables such as an extensive list of clinical sites or the projected subject accrual and were excluded in descriptive statistics. Second, this study is limited to OM and warrants further investigation into whether our conclusions are observed in other oral complications secondary to cancer therapy, such as jaw osteonecrosis and xerostomia.

The features of successful OM CTs are complex and frequently cited reasons for failure include achieving optimal trial participation, demonstrating clinical efficacy and safety, and avoiding commercial or site issues, among others. Improvement of recruitment/enrollment is not trivial; however, potential solutions include the incorporation of automated referral systems for trial enrollment [11], and improved promotion of open CTs [12]. It has been observed that the utility of a biomarker for the selection of subjects improves the probability of success, supporting the need for biomarkers for OM development [13]. To address safety/efficacy challenges, numerous strategies have been described to minimize treatment-related toxicities such as the inclusion of patient-reported outcomes [14], toxicogenomic analysis [15], and integration of computational methods such as AlphaFold into mechanistic studies [16]. Future OM trials ought to incorporate blinded study design and improved strategies to improve subject accrual. In agreement with our data, a recent report comparing successful and failed phase III trials for the treatment of OM revealed differences in sponsor funding and patient inclusion criteria [10]. Our findings across all phases suggest increased attention ought to be given to sponsorship selection, since on average, completed trials had fewer collaborative groups and private sponsors and a greater proportion of industry sponsors across all phases, in comparison to matched failed trials. Taken together, it is important that funding organizations and investigators incorporate study characteristics of both failed and completed OM CTs, towards the goal of achieving an effective treatment for OM.

## Supporting information

Reznik et al. supplementary material Reznik et al. supplementary material

## Supplementary material

The supplementary material for this article can be found at https://doi.org/10.1017/cts.2025.65
