# Supplementary material for: Characterization of terminated and withdrawn clinical trials for the treatment and prevention of oral mucositis
Source: J Clin Transl Sci. 2025 Apr 10;9(1):e86. doi: 10.1017/cts.2025.65 (PMC12083202; doi:10.1017/cts.2025.65)
Supplement: Reznik et al. supplementary material [file S2059866125000652sup001.docx]

## **Supplementary Information**

## Characterization of terminated and withdrawn clinical trials for the treatment and prevention of oral mucositis

Alex Reznik^1^, Stephen Sonis, Alessandro Villa

^1^Medical Scientist Training Program (MSTP), University of Miami Miller School of Medicine, 1600 NW 10th Ave, Miami, FL 33136

[asr2224@med.miami.edu](mailto:asr2224@med.miami.edu)

**Supplementary Table 1.** Additional characteristics of failed oral mucositis clinical trials.

| **NCT Identifier** | **Phase** | **Projected Accrual** | **Actual Accrual** | **% Accrual^a^** | **# Sites** | **Reasons for Failure** | **Comments** |
| --- | --- | --- | --- | --- | --- | --- | --- |
| **Terminated:** |  |  |  |  |  |  |  |
| NCT00427102 | 1 | NR | NR | - | NR | Investigator/Site Issues | No subjects enrolled and PI went to another facility |
| NCT01092975 | 1 | 51 | 1 | 2.0% | 1 | Other | Formulation/dose changes; planned changes to safety monitoring/reporting |
| NCT01975688 | 1 | NR | 10 | - | 1 | Recruitment/Enrollment | Slow recruitment |
| NCT00352118 | 2 | NR | 4 | - | 1 | Efficacy/Safety | Low dose radiation treatment was not appropriate for these patients. |
| NCT00503776 | 2 | 60 | 41 | 68.3% | 1 | Funding/Sponsorship | Funding became unavailable |
| NCT00947466 | 2 | 60 | 25 | 41.7% | 2 | Efficacy/Safety | 25 patients have been recruited and it was considered that further recruitment would add no extra PK information |
| NCT01403064 | 2 | 96 | 76 | 79.2% | 31 | Efficacy/Safety | Due to lack of efficacy |
| NCT01682031 | 2 | NR | 18 | - | 2 | Funding/Sponsorship | Due to a lack of funding |
| NCT02300727 | 2 | 38 | 6 | 15.8% | 1 | Recruitment/Enrollment | Protocol failed to accrue sufficient subject to complete meaningful analysis. |
| NCT02542215 | 2 | 285 | 47 | 16.5% | 76 | Efficacy/Safety | https://www.biospace.com/article/releases/sucampo-terminates-development-of-cobiprostone-following-phase-ii-data-/ |
| NCT02575313 | 2 | NR | 10 | - | 2 | Recruitment/Enrollment | NR |
| NCT02575391 | 2 | NR | 7 | - | 2 | Recruitment/Enrollment | Lack of participant recruitment |
| NCT02606994 | 2 | NR | 1 | - | 1 | Recruitment/Enrollment | Lack of enrollment |
| NCT03234465 | 2 | 200 | 200 | 100% | 50 | Efficacy/Safety | No efficacy could be demonstrated, sponsor decided to prematurely end the study. |
| NCT03602066 | 2 | NR | 14 | - | 1 | Recruitment/Enrollment | Study terminated by pharmaceutical company |
| NCT04239261 | 2 | 50 | 10 | 20.0% | 1 | Recruitment/Enrollment | Recruitment slower than anticipated |
| NCT00006994 | 3 | 158 | 23 | 14.6% | 106 | Funding/Sponsorship | Withdrawn due to drug availability and funding |
| NCT00224692 | 3 | 50 | 50 | 100% | 1 | Undisclosed | NR |
| NCT00360971 | 3 | 298 | 21 | 7.0% | 48 | Other | Due to positive preliminary results from other palifermin studies. |
| NCT00574860 | 3 | 240 | 240 | 100% | 2 | Other | Additional research |
| NCT01066741 | 3 | 330 | 78 | 23.6% | 1 | Recruitment/Enrollment | Insufficient recruitment, the planned sample size appears not achievable |
| NCT02539342 | 3 | NR | 1 | - | 1 | Funding/Sponsorship | Lack of funding |
| NCT04648020 | 3 | 260 | 190 | 73.1% | 74 | Efficacy/Safety | Interim Analysis did not meet the pre-defined threshold for efficacy of a 15% absolute difference in SOM prevention between Validive and placebo. |
| NCT03490396 | 4 | NR | 28 | - | 2 | Recruitment/Enrollment | NR |
| NCT01883908 | N/A | NR | 4 | - | 1 | Investigator/Site Issues | The project is terminated due to that fact that the PI has moved to another institution and there are no resources to keep the study open in either institution. |
| NCT02326675 | N/A | NR | 29 | - | 1 | Undisclosed | NR |
| NCT03843554 | N/A | 120 | 60 | 50.0% | 1 | Recruitment/Enrollment | Study was terminated with 50% enrollment by the sponsor due to under enrollment into specific diverse racial, ethnic, and sex/gender categories |
| NCT04321850 | N/A | 108 | 21 | 19.4% | 1 | Other | Health restrictions due to the COVID-19 pandemic |
| NCT00176514 | Pilot | NR | 37 | - | 1 | Recruitment/Enrollment | Slow accrual |
| **Withdrawn:** |  |  |  |  |  |  |  |
| NCT01728480 | 1 | NR | 0 | 0% | NR | Funding/Sponsorship | Financial Sponsor requested termination |
| NCT01820091 | 1 | NR | 0 | 0% | NR | Undisclosed | NR |
| NCT05104268 | 1 | NR | 0 | 0% | 1 | Recruitment/Enrollment | Unable to recruit due to the relatively infrequent occurrence of oral mucositis |
| NCT00929825 | 1 | 60 | 0 | 0% | 1 | Investigator/Site Issues | The Hospital had the Unit of Stem Cell Transplantation suspended |
| NCT00540332 | 2 | 40 | 0 | 0% | NR | Efficacy/Safety | The 20070201 study was terminated based on evaluation of Palifermin solid tumor data. |
| NCT01674374 | 2 | NR | 0 | 0% | NR | Undisclosed | NR |
| NCT03982537 | 2 | NR | 0 | 0% | NR | Other | Concept is withdrawn and a different concept will be submitted the near future. |
| NCT05331131 | 2 | NR | 0 | 0% | 1 | Other | Study team not moving forward with protocol |
| NCT03955224 | 2 | NR | 0 | 0% | NR | Recruitment/Enrollment | Withdrawn due to lack of recruitment |
| NCT00162526 | 2 | 30 | 0 | 0% | 1 | Investigator/Site Issues | The PI is no longer work at Hadassah |
| NCT01797952 | 3 | NR | 0 | 0% | 1 | Investigator/Site Issues | PI was away on sabbatical for 2 years. Protocol is being revised before starting the study. |
| NCT01545687 | 3 | NR | 0 | 0% | NR | Undisclosed | NR |
| NCT00474110 | 3 | 20 | 0 | 0% | NR | Recruitment/Enrollment | Clinical practice had changed between time of initial protocol development and subject recruitment. We were not able to find eligible patients. |
| NCT01288625 | 4 | NR | 0 | 0% | NR | Funding/Sponsorship | There were IP availability issues due to which the study could not be started. |
| NCT00549835 | N/A | NR | 0 | 0% | 1 | Funding/Sponsorship | Inadequate funding |
| NCT00334984 | N/A | NR | 0 | 0% | NR | Funding/Sponsorship | Withdrawn due to inability to reach an acceptable agreement with industry sponsor |
| NCT00928161 | N/A | NR | 0 | 0% | NR | Undisclosed | NR |

Abbreviations: NR, not reported; N/A, not applicable; PI, principal investigator; PK, pharmacokinetics; SOM, severe oral mucositis; IP, intellectual property

^a^The denominator is projected accrual and the nominator is actual accrual
